# Disruption of CTCF at the *miR-125b1* locus in gynecological cancers and breast cancer cell lines

**DOI:** 10.1186/1756-8935-6-S1-P79

**Published:** 2013-03-18

**Authors:** Ernesto Soto-Reves, Fernanda Cisneros-Soberanis, Iliana Alcalá Moreno, Rodrigo Gonzaílez Barrios, David Cantú de León, Clementina Castro, Luis A Herrera

**Affiliations:** 1 Unidad de Investigacioín Biomeídica en Caíncer- Instituto National de Cancerologiía -Instituto de Investigaciones Biomeídicas, Universidad Nacional Autoínoma de Meíxico; D. F. Meíxico

## Background

In cancer cells, transcriptional gene silencing has been associated with genetic and epigenetic defects. The disruption of DNA methylation patterns and covalent histone marks has been associated with cancer development. In non-neoplastic cells the multifunctional CCCTC-binding factor (CTCF) can serve as a barrier against the spread of DNA methylation and histone repressive marks into promoter regions of tumor suppressor genes such as *BRCA1*, *Rb*, *p16* and *p53*. The absence of CTCF has been related with its epigenetic gene silencing. Until recently, microRNA (miRNA) gene silencing was not well understood. In particular, *miR- 125b1* has been suggested to be a miRNA with tumor suppressor activity, and it has been shown to be deregulated in various human cancers.

## Materials and methods

As a first approach we performed a functional promoter characterization of a CpG island proximal to the transcription star site of the *miR-125b1* gene. After, demonstrating the promoter activity, we focused in evaluating the DNA methylation of this region in cancer cell lines as well as in normal tissues and gynecological tumor samples. We determine the effect of DNA methylation at the CpG island *of miR-125b1* on the expression of this gene, we performed a qRT-PCR assay. In addition, we analyzed the association of CTCF and covalent histone modifications at the *miR-125b1* locus.

## Results

Our results demonstrated the dissociation of CTCF at the *miR-125b1* gene promoter in breast cancer cell lines and in gynecological cancers. The disruption of CTCF in breast cancer cells correlated with the incorporation of repressive histone marks such H3K9me3 and H3K27me3 as well as with aberrant DNA methylation patterns. We observed a significant reduction on the expression of *miR-125b1* in cancer cells in comparison with controls, suggesting that DNA methylation and repressive histone marks at the CpG island might reduce *miR-125b1* expression. These effects were observed in other gynecological cancers, including ovarian and cervical tumors.

## Conclusions

A reduction *of miR-125b1* expression in cancers, correlated with methylation, repressive histone marks and loss of CTCF binding at the promoter region.

